# Declining tobacco control awareness and support among Polish adolescents: Trends from the Global Youth Tobacco Survey 2003–2022

**DOI:** 10.18332/tpc/208451

**Published:** 2025-08-21

**Authors:** Paweł Koczkodaj, Irmina M. Michałek

**Affiliations:** 1 Cancer Epidemiology and Primary Prevention Department, Maria Sklodowska-Curie National Research Institute of Oncology, Warsaw, Poland; 2 Department of Cancer Pathology, Maria Sklodowska-Curie National Research Institute of Oncology, Warsaw, Poland

**Keywords:** tobacco, nicotine, adolescents, youth, awareness, Poland

## Abstract

**INTRODUCTION:**

The Global Youth Tobacco Survey (GYTS) is a cross-sectional, schoolbased survey that provides insight into tobacco use and related behaviors among adolescents aged 13–15 years. This study examines trends in tobacco-related attitudes, education, and media exposure among Polish youth, using data from the 2022 GYTS alongside previous surveys conducted in 2003 and 2016.

**METHODS:**

The study sample was stratified by geographical regions: rural areas, small towns, and large cities. Data were collected from 98 schools across Poland, involving 95 schools (96.9%), 224 classes (97.8%), and 3985 students (78.9%). The primary analysis focused on 3573 students aged 13–15 years. Data from 2003 and 2016 GYTS editions were also used for comparison. Sampling, data weighting, and the methodological framework followed WHO guidelines.

**RESULTS:**

Between 2003 and 2022, there was a notable decline in the proportion of Polish adolescents receiving tobacco education in school, from 61.4% to 43.1%. Perceptions of the harms of secondhand smoke also fell substantially, from 65.8% to 34.4%. Support for smoke-free policies weakened, with a decline in support for both indoor and outdoor smoking bans. Exposure to anti-tobacco messages in media dropped dramatically, from 89.4% in 2003 to 34.9% in 2022. On the other hand, perceptions that smoking enhances social interactions rose from 40.8% to 45.5%, while tobacco industry advertising and depictions of tobacco use in media remained prevalent, though declining over time.

**CONCLUSIONS:**

The findings suggest concerning trends in tobacco-related attitudes and behaviors among Polish adolescents, indicating weakened prevention efforts and a shift towards pro-tobacco norms. The decline in tobacco education, public support for smoke-free policies, and media exposure to anti-tobacco messages highlight the need for renewed public health interventions. Strengthening school-based education, reinforcing smoke-free policies, and regulating tobacco portrayals in media are critical to reversing these trends. Additionally, policy measures such as plain packaging and a ban on tobacco displays at points of sale are necessary to protect future generations from tobacco initiation. Without decisive action, there is a risk of undermining the progress made in tobacco control.

## INTRODUCTION

It is estimated that in Poland, tobacco smoke is responsible for two-thirds of all cancer-related deaths^1^. Poland is among a small group of European Union (EU) countries with the highest rate of years lost due to premature death or disability caused by tobacco consumption – with a disability-adjusted life years (DALY) burden ranging from 17.5% to <20%^2^. In this context, efforts aimed at eliminating this risk factor remain a key priority for improving public health in Poland.

Countries such as New Zealand, certain regions of the United States, and Australia have adopted first tobacco-free generation strategies, gradually phasing out tobacco products to protect public health, particularly among younger populations^3^. A similar approach has been incorporated into the Europe’s Beating Cancer Plan, which envisions reducing tobacco use across the EU to below 5% by 2040, compared to the current 25%^4^. A study published in 2024 by the International Agency for Research on Cancer (IARC) in The Lancet Public Health suggests that banning the sale of tobacco products to individuals born between 2006 and 2010 could prevent 1.2 million lung cancer deaths worldwide by 2095 across 185 countries^5^.

Against the backdrop of tobacco consumption in Poland and the increasing use of new nicotine products (heated tobacco, e-cigarettes), a particularly alarming trend is the high level of interest in these products among children and adolescents. According to national studies, the prevalence of cigarette ever use among youth and young adults aged 13–19 years is 32%, with nearly 61% of them susceptible to e-cigarette use^6^. Beyond their inherent health risks, these novel tobacco and nicotine products often serve as a gateway to traditional cigarette smoking, particularly for young people^7^.

One of the most influential factors shaping youth attitudes toward tobacco and new nicotine products is marketing exposure. Despite a nationwide ban on the promotion and advertising of these products in Poland, research conducted in 2021 found that various forms of marketing remain prevalent at points of sale, including those located near schools^8^. Without stronger educational efforts to counter these influences, the ambition of creating the first tobacco-free generation in Poland – and across EU – could be irreversibly undermined.

## METHODS

The Global Youth Tobacco Survey (GYTS) follows a cross-sectional, nationally representative, school-based survey approach, targeting students aged 13–15 years. A detailed description of the methodology, standardized for all countries participating in the study, is available on the World Health Organization (WHO) website^9^.

In our study, the sample was structured by dividing the population into three main categories: rural areas (villages), small towns (urban or urban-rural areas with fewer than 5000 residents and city status), and large cities (urban or urban-rural areas with city status and more than 5000 residents). The national dataset was created by combining the data from all three areas. To maintain representativeness, separate sampling and data weighting were applied to each of these regions. The sampling frame consisted of schools that offered education for 7th and 8th-grade elementary students and 1st-grade high school students, with a minimum enrollment of 40 students. In the 2022 GYTS edition, 98 schools from various regions of Poland took part.

The 2022 GYTS in Poland achieved an overall response rate of 74.8%, demonstrating strong participation at all levels of sampling. Of the 98 schools initially selected, 95 (96.9%) participated in the survey. Additionally, 224 out of 229 classes (97.8%) were involved, and 3985 out of 5049 sampled students (78.9%) responded. The primary analysis focused on 3573 students aged 13–15 years, providing a comprehensive look into tobacco-related behaviors within this age group.

For comparison, the results from the 2022 GYTS were analyzed together with data from the previous Polish surveys conducted in 2003 and 2016 which followed the same standardized WHO methodology^10,11^.

## RESULTS

### Trends in attitudes and tobacco education among Polish youth

Between 2003 and 2022, there was a notable decline in the proportion of Polish adolescents who reported being taught in school about the dangers of tobacco use (Table 1). In 2003, 57.3% (95% CI: 52.8–61.6) of youth overall reported receiving tobacco education in school, increasing to 61.4% (95% CI: 58.1–64.7) in 2016, but subsequently dropping to 43.1% (95% CI: 40.0–46.2) in 2022. This decline was observed across both sexes, with males decreasing from 55.5% (95% CI: 50.5–60.4) in 2003 to 42.3% (95% CI: 38.6–46.1) in 2022, and females from 59.6% (95% CI: 54.3–64.7) to 44.1% (95% CI: 40.6–47.8).

**Table 1:** Trends in attitudes and tobacco education among Polish youth (2003, 2016, 2022)

*Items*	*Overall*			*Males*			*Females*		
	*2003*	*2016*	*2022*	*2003*	*2016*	*2022*	*2003*	*2016*	*2022*
Taught in school about the dangers of tobacco use	57.3 (52.8–61.6)	61.4 (58.1–64.7)	43.1 (40.0–46.2)	55.5 (50.5–60.4)	56.7 (52.8–60.6)	42.3 (38.6–46.1)	59.6 (54.3–64.7)	66.1 (62.1–69.9)	44.1 (40.6–47.8)
Definitely thought other people’s tobacco smoking is harmful to them	65.8 (62.5–68.9)	49.6 (47.5–51.7)	34.4 (30.9–38.0)	63.6 (60.1–66.9)	48.5 (45.4–51.5)	37.6 (33.3–42.1)	67.9 (63.4–72.2)	50.7 (47.4–54.0)	30.7 (27.2–34.5)
Definitely thought it is difficult to quit once someone starts smoking tobacco	-	29.9 (28.2–31.8)	31.5 (28.5–34.7)	-	26.9 (24.1–29.8)	30.1 (26.3–34.2)	-	33.1 (30.6–35.7)	33.1 (29.2–37.3)
Thought smoking tobacco helps people feel more comfortable at celebrations, parties, and social gatherings	-	40.8 (38.9–42.7)	45.5 (42.8–48.3)	-	40.3 (37.6–43.1)	46.2 (43.1–49.4)	-	41.1 (38.7–43.6)	44.8 (40.3–49.4)
Favored banning smoking inside enclosed public places	75.0 (72.7–77.1)	74.7 (72.4–76.8)	69.6 (66.1–72.8)	74.7 (71.5–77.7)	71.0 (67.9–73.9)	67.1 (62.9–71.1)	75.7 (72.3–78.7)	78.4 (75.8–80.7)	72.1 (68.0–75.9)
Favored banning smoking at outdoor public places	-	54.6 (52.4–56.8)	45.3 (42.0–48.7)	-	55.0 (51.4–58.5)	47.7 (43.5–51.9)	-	54.3 (51.3–57.3)	42.7 (38.8–46.7)

All values represent the percentage of respondents who selected the given response, with 95% confidence intervals provided in parentheses. A hyphen (‘-’) indicates that the item was not included in the respective edition of the Global Youth Tobacco Survey (GYTS).

Perceptions of secondhand smoke as harmful also declined substantially. In 2003, 65.8% (95% CI: 62.5–68.9) of adolescents strongly agreed that exposure to others’ tobacco smoke was harmful, but this fell to 49.6% (95% CI: 47.5–51.7) in 2016 and further to 34.4% (95% CI: 30.9–38.0) in 2022. The decline was more pronounced among females, decreasing from 67.9% (95% CI: 63.4–72.2) in 2003 to just 30.7% (95% CI: 27.2–34.5) in 2022.

Beliefs about the addictiveness of smoking showed little change over time. In 2016, 29.9% (95% CI: 28.2–31.8) of respondents agreed that quitting smoking is difficult, compared to 31.5% (95% CI: 28.5–34.7) in 2022.

Notably, the perception that smoking enhances social interactions increased over time. In 2016, 40.8% (95% CI: 38.9–42.7) of youth believed that smoking tobacco made social gatherings more comfortable; by 2022, this had risen to 45.5% (95% CI: 42.8–48.3). The trend was consistent among both sexes.

Support for smoke-free policies also declined. In 2003, 75.0% (95% CI: 72.7–77.1) of youth supported banning smoking in enclosed public spaces. While this remained relatively stable in 2016 (74.7%; 95% CI: 72.4–76.8), it decreased to 69.6% (95% CI: 66.1–72.8) in 2022. Support for outdoor smoking bans followed a similar trend, dropping from 54.6% (95% CI: 52.4–56.8) in 2016 to 45.3% (95% CI: 42.0–48.7) in 2022.

### Exposure to protobacco and anti-tobacco messaging

Exposure to anti-tobacco messaging in the media declined significantly over time (Table 2). In 2003, 89.4% (95% CI: 88.0–90.7) of adolescents who consumed media reported seeing anti-tobacco messages. This dropped sharply to 43.2% (95% CI: 41.2–45.3) in 2016 and further to 34.9% (95% CI: 32.1–37.7) in 2022. Exposure was comparable between males and females.

**Table 2:** Exposure to pro- and anti-tobacco messaging among Polish youth (2003, 2016, 2022)

*Items*	*Overall*			*Males*			*Females*		
	*2003*	*2016*	*2022*	*2003*	*2016*	*2022*	*2003*	*2016*	*2022*
**Anti-tobacco advertising**									
Anti-tobacco messages in the media (Among those who used media in the past 30 days)	89.4 (88.0–90.7)	43.2 (41.2–45.3)	34.9 (32.1–37.7)	87.9 (86.1–89.6)	41.8 (39.2–44.5)	34.5 (31.9–37.2)	90.5 (88.3–92.4)	44.6 (41.6–47.6)	35.1 (30.8–39.6)
Anti-tobacco messages at sporting or community events (Among those who attended those events in the past 30 days)	46.2 (43.7–48.8)	24.6 (21.7–27.7)	30.9 (27.9–34.0)	46.6 (42.7–50.6)	28.4 (24.6–32.5)	31.7 (27.7–36.0)	45.3 (42.3–48.4)	20.1 (17.3–23.1)	29.7 (26.2–33.5)
**Tobacco industry advertising**									
Noticed tobacco advertisements or promotions at points of sale (Among those who visited a point of sale in the past 30 days)	-	43.3 (40.7–45.9)	29.9 (27.6–32.3)	-	43.1 (40.2–46.0)	30.3 (27.6–33.2)	-	43.4 (39.6–47.2)	29.2 (26.6–31.9)
Noticed anyone using tobacco on television, videos, or movies (Among those who watched television, videos, or movies in the past 30 days)	93.2 (91.6–94.6)	71.9 (69.9–73.8)	61.9 (59.1–64.6)	92.4 (90.3–94.1)	70.7 (67.5–73.6)	62.2 (59.0–65.4)	94.0 (91.8–95.7)	73.1 (70.3–75.6)	61.5 (57.6–65.3)
Ever offered a free tobacco product from a tobacco company representative	25.7 (23.4–28.2)	6.0 (4.9–7.3)	6.8 (5.6–8.2)	29.3 (26.1–32.8)	7.7 (6.1–9.7)	8.2 (6.4–10.6)	22.7 (19.8–25.8)	4.2 (3.2–5.5)	5.2 (4.1–6.5)
Owned something with a tobacco brand logo on it	26.5 (23.9–29.3)	11.9 (10.3–13.8)	8.6 (7.2–10.2)	29.5 (26.7–32.5)	15.2 (12.6–18.4)	9.3 (7.2–11.8)	23.7 (19.9–27.9)	8.6 (7.3–10.2)	7.7 (6.0–9.7)

All values represent the percentage of respondents who selected the given response, with 95% confidence intervals provided in parentheses. A hyphen (‘-’) indicates that the item was not included in the respective edition of the Global Youth Tobacco Survey (GYTS).

Similarly, anti-tobacco messaging at community or sporting events declined from 46.2% (95% CI: 43.7–48.8) in 2003 to 24.6% (95% CI: 21.7–27.7) in 2016, before slightly rebounding to 30.9% (95% CI: 27.9–34.0) in 2022.

Conversely, exposure to tobacco industry advertising remained a concern. In 2016, 43.3% (95% CI: 40.7–45.9) of adolescents who had visited a point of sale in the past 30 days reported seeing tobacco advertisements, decreasing to 29.9% (95% CI: 27.6–32.3) in 2022.

Depictions of tobacco use in entertainment media remained widespread but declined over time. In 2003, 93.2% (95% CI: 91.6–94.6) of youth reported noticing tobacco use in television, videos, or movies, decreasing to 71.9% (95% CI: 69.9–73.8) in 2016 and further to 61.9% (95% CI: 59.1–64.6) in 2022.

Direct marketing from the tobacco industry has become less prevalent. The proportion of youth who had ever been offered a free tobacco product from a company representative declined markedly from 25.7% (95% CI: 23.4–28.2) in 2003 to 6.0% (95% CI: 4.9–7.3) in 2016 and remained low in 2022 (6.8%, 95% CI: 5.6–8.2). Similarly, youth ownership of items featuring tobacco branding declined from 26.5% (95% CI: 23.9–29.3) in 2003 to 8.6% (95% CI: 7.2–10.2) in 2022.

## DISCUSSION

According to the announcements of the Ministry of Education in Poland, starting in September 2025, health education will replace family life education as an elective subject^12^. This course will be offered to students in grades 4–8 of primary schools and grades 1–3 of secondary schools, including general high schools, technical schools, and first-level vocational schools. Considering the findings of our study, this direction appears to be fully justified. Over the analyzed period (2016–2022), we observed a significant decline in the proportion of adolescents aged 13–15 years who received school-based education on the health hazards of tobacco use, with only 43% of respondents in 2022 reporting such instruction. Undoubtedly, schools should serve as a key setting for delivering reliable information about both traditional tobacco products and emerging nicotine-containing alternatives. Alarmingly, health data and successive GYTS findings highlight the necessity of seriously considering making this subject mandatory to achieve a satisfactory population-level impact of the implemented educational efforts in the coming decades. The effectiveness of school-based education in tobacco and nicotine prevention is further supported by pilot programs and available educational tools, including online resources^13^. Therefore, integrating comprehensive tobacco education that addresses all forms of tobacco and nicotine use is crucial.

Throughout the analyzed period, we also observed a steady decline in adolescents’ perception of secondhand smoke as a health hazard. However, a study conducted by Song et al.^14^ demonstrated that the perception of secondhand smoke exposure is strongly associated with smoking initiation. Adolescents who recognized personal exposure to tobacco smoke as a health risk exhibited significantly lower likelihoods of initiating smoking^14^. Furthermore, our study revealed that the decline in awareness was more pronounced among girls, aligning with previous research findings that emphasize the significant role of gender in youth smoking behaviors^15^.

Another notable trend identified in our study is the increasing perception of tobacco use as a means of facilitating social interactions among adolescents. This observation is consistent with prior research underscoring the importance of social context in smoking behaviors, particularly among younger age groups. A systematic review by Littlecott et al.^16^ highlights the substantial influence of peer groups on adolescent tobacco use. This effect is further amplified when individuals who are most socially influential within their peer networks engage in smoking behaviors^17^. The persistent popularity of tobacco products among Polish youth presents an unfavorable outlook for reducing smoking prevalence not only among younger age groups but also within the broader adult population in Poland.

The findings on the reach of anti-tobacco messages to Polish youth through the media are highly concerning. Our study showed that over the years, the exposure of young people to media messages about the harmful effects of tobacco has significantly declined. The same trend was observed with respect to local and sports events. On the other hand, exposure to tobacco-related promotional activities and advertisements at points of sale – despite the ban in Poland – remains relatively high (around 30% of adolescents who had visited a point of sale in the past 30 days reported seeing tobacco advertisements in 2022). Research conducted by Koczkodaj et al.^8^ similarly showed that, despite the legal ban, 76% of the retail points near secondary schools in Warsaw exposed customers to various forms of cigarette, e-cigarette, and HTP advertising, violating Polish law. Another study conducted in Gdańsk, Poland, found that tobacco product advertising was present in two-thirds of the surveyed nightclubs^18^. Additionally, research by Polanska et al.^19^ highlights the poor enforcement of the ban on tobacco and e-cigarette advertising in Poland, which provides the tobacco industry with an opportunity to promote their products through unlawful means.

An important and still unresolved issue in Poland is the depiction of tobacco use in entertainment media. Our study indicates that while this phenomenon is weakening, it remains a significant challenge. In 2022, nearly 62% of respondents noticed such tobacco product promotions. This is corroborated by a study conducted by Rath et al.^20^, which found that 86% of Netflix programs and 86% of broadcast and cable TV programs featured at least one instance of tobacco use. This analysis covered the 2015–2016 and 2016–2017 seasons, examining 14 programs aired on the Netflix streaming platform as well as on broadcast and cable television^20^. Meanwhile, as noted by the Surgeon General, there is a causal link between the portrayal of smoking in films and the initiation of smoking among young individuals^21^.

### Strengths and limitations

GYTS employs a standardized methodology, ensuring consistency and comparability across international studies. In the 2022 GYTS conducted in Poland, the survey achieved high participation rates, with 96.9% of sampled schools and 97.8% of selected classes participating. School enrollment for adolescents aged 13–15 years in Poland has remained consistently very high across all survey years, minimizing potential bias from the exclusion of out-of-school youth. This high level of engagement enhances the reliability and generalizability of the findings. The use of a questionnaire covering various topics, including tobacco use (smoking and smokeless), cessation, secondhand smoke exposure, and pro- and anti-tobacco advertising and promotion, allows for comprehensive data collection.

However, certain limitations should be considered. The reliance on self-reported data may introduce reporting biases, such as underreporting or overreporting of tobacco use behaviors, due to social desirability or recall biases. Additionally, the cross-sectional design of the survey captures data at a single point in time, limiting the ability to infer causal relationships or assess changes in behaviors over time.

## CONCLUSIONS

This study reveals concerning trends in tobacco-related attitudes, education, and policy support among Polish adolescents. The proportion of youth receiving school-based tobacco education dropped from 61.4% in 2016 to 43.1% in 2022, while the share recognizing secondhand smoke as harmful fell from 49.6% to just 34.4% over the same period. These declines indicate weakening prevention efforts. At the same time, the rising perception that smoking enhances social interactions (from 40.8% to 45.5%) underscores the need to counteract pro-tobacco norms.

Support for smoke-free policies has also weakened. While 75.0% of youth favored banning smoking indoors in 2003, this fell to 69.6% in 2022. Outdoor smoking bans saw an even steeper decline, from 54.6% in 2016 to 45.3% in 2022. As public endorsement is key to effective policy enforcement, efforts should reinforce the benefits of smoke-free environments.

Meanwhile, exposure to anti-tobacco messaging in media plummeted from 89.4% in 2003 to just 34.9% in 2022. To prevent a reversal in tobacco control progress, policymakers must reinvest in school-based education, strengthen smoke-free policies, revitalize public health campaigns, and regulate tobacco portrayals in media.

There are systemic measures – aligned with the WHO Framework Convention on Tobacco Control, to which Poland is a signatory – that remain awaiting implementation yet could play a crucial role in preventing the youngest generations from initiating tobacco and nicotine use^22^. Chief among these are plain packaging and a complete ban on the display of tobacco and nicotine products at points of sale. Moreover, strengthening age verification at points of sale, as well as implementing a comprehensive ban on the online sale of all tobacco and nicotine products, appears to be both justified and urgently needed.

Without further decisive action from policymakers in this regard, negative trends may jeopardize the progress achieved in public health.

## Data Availability

The data supporting this research are available from the following sources: https://www.who.int/teams/noncommunicable-diseases/surveillance/systems-tools/global-youth-tobacco-survey
